# Factors Associated With Glycemic Control During Free-Living Overnight Closed-Loop Insulin Delivery in Children and Adults With Type 1 Diabetes

**DOI:** 10.1177/1932296815604439

**Published:** 2015-10-30

**Authors:** Martin Tauschmann, Hood Thabit, Lalantha Leelarathna, Daniela Elleri, Janet M. Allen, Alexandra Lubina-Solomon, Marietta Stadler, Emma Walkinshaw, Ahmed Iqbal, Pratik Choudhary, Malgorzata E. Wilinska, Simon R. Heller, Stephanie A. Amiel, Mark L. Evans, David B. Dunger, Roman Hovorka

**Affiliations:** 1 Wellcome Trust-MRC Institute of Metabolic Science, University of Cambridge, Cambridge, UK; 2 Academic Unit of Diabetes, Endocrinology and Metabolism, Department of Human Metabolism, University of Sheffield, Sheffield, UK; 3 Diabetes Research Group, King’s College London, London, UK

**Keywords:** closed-loop insulin delivery, glycemic control, home study, predictive factors, type 1 diabetes

Unsupervised free-living overnight home use of closed-loop insulin delivery is feasible, safe, and effective in adolescents^1^ and adults^2^ with type 1 diabetes, but outcomes vary between individuals. Understanding factors influencing glucose outcomes may help to identify vulnerable populations, guide design of future studies, and lead to enhanced control algorithms.

To explore associations between demographic characteristics, the use of closed-loop and glucose performance, we pooled data from 2 multicenter trials, 1 involving adolescents,^1^ and 1 involving adults^2^ with type 1 diabetes. Both studies adopted an open-label, cross-over, randomized controlled study design. Participants were randomly assigned to 4 (adults) or 3 (adolescents) weeks of sensor-augmented pump therapy with or without overnight closed-loop. An identical model-predictive-control algorithm was used in both studies.^3^ Participants were instructed to start the system at home after their evening meal and to discontinue it before breakfast the next morning. Detailed methods and results are reported elsewhere.^1-2^

In the present work, Pearson’s correlation coefficients quantified the relationship between baseline demographic factors (age, BMI, HbA1c, total daily dose), participant-level utility characteristics (average duration of closed-loop application, average start time of closed-loop) and closed-loop outcomes between midnight and 08:00 (mean glucose, time in target between 70 and 145 mg/dl, time below 70 mg/dl) (Table 1). Age and time below target were rank-normal transformed. Associations with gender were evaluated applying Spearman correlation. Multiple linear regression analysis quantified the amount of explained variability of closed-loop outcomes using demographic and utility characteristics.

**Table 1.** Pearson’s Correlation Coefficients Between Closed-Loop Outcomes and Demographic and Utility Characteristics (N = 40).

	Age	BMI	HbA1c	Total daily dose	Duration of closed-loop application	Time of closed-loop start
Mean glucose (*P* value)	.17 (.294)	.10 (.550)	.52 (.001)	−.25 (.119)	−.20 (.209)	.25 (.117)
Time in target 70-145 mg/dl (*P* value)	−.33 (.038)	−.24 (.129)	−.26 (.101)	.27 (.097)	.30 (.064)	−.30 (.064)
Time below 70 mg/dl (*P* value)	.04 (.786)	.14 (.386)	−.43 (.006)	.06 (.702)	−.12 (.473)	−.25 (.127)

Forty participants completed the studies, including 24 adults (age 43 ± 12 years [mean ± SD]; HbA1C 64.9 ± 8.9mmol/mol, 8.1 ± 0.8%; BMI 26.0 ± 3.5kg/m^2^; total daily insulin dose 0.5 ± 0.1U/kg/day) and 16 adolescents (age 15.6 ± 2.1 years; HbA1C 63.9 ± 9.4mmol/mol, 8.0 ± 0.9%; BMI 22.4 ± 3.7kg/m2; total daily insulin dose 0.8 ± 0.2U/kg/day).

Data on 866 closed-loop nights were analyzed. HbA1c at baseline was associated with mean glucose during closed-loop nights (*r* = .52, *P* = .001) and time with hypoglycemia (*r* = –.43, *P* = .006), but not time in target (*r* = –.26, *P* = .101). Early closed-loop start and longer closed-loop application tended to increase time in target (*P* = .064). There was an age-associated reduction in time in target (*r* = –.33, *P* = .038), perhaps reflecting the association between older age and shorter period of closed-loop use (*r* = –.58, *P* < .001). Of the variance in mean glucose, 33% was explained by the regression model (*P* = .028), with HbA1c as the only significant predictor (*P* = .001). For time below target, the explained variance was 36% (*P* = .017); earlier closed-loop start time (*P* = .017) and HbA1c (*P* = .008) were significant predictors. Only 20% of variance in time in target was explained by the regression model.

The strength of the current work is that the data were collected during free-living unsupervised home closed-loop use. Weaknesses include that we did not capture at all or with low confidence other potentially influential factors such as socioeconomic and educational status, exercise patterns, and meal size and composition.

In conclusion, in adolescents and adults with type 1 diabetes undergoing overnight closed-loop, baseline HbA1c is correlated with mean overnight glucose but not time in target range. Despite closed-loop, a lower HbA1c level remains a risk factor for nocturnal hypoglycemia. Improved time in target may be observed if overnight closed-loop is started earlier and applied for longer.
